# Sequencing Alpha-1 MZ Individuals Shows Frequent Biallelic Mutations

**DOI:** 10.1155/2018/2836389

**Published:** 2018-09-05

**Authors:** Kimberly E. Foil, M. Gwen Blanton, Chris Sanders, Joannah Kim, Haitham S. Al Ashry, Suchit Kumbhare, Charlie Strange

**Affiliations:** ^1^Division of Pulmonary, Critical Care, Allergy and Sleep Medicine, Medical University of South Carolina, Charleston, SC, USA; ^2^Biocerna LLC, Gaithersburg, MD, USA

## Abstract

**Rationale:**

Individuals with a single Z mutation in the* SERPINA1* gene that codes for alpha-1 antitrypsin (AAT) are at increased risk for COPD if they have ever-smoked. Whether additional variants alter the risk for COPD in this population remains unknown.

**Objectives:**

To determine whether additional* SERPINA1* variants impact COPD development in a previously identified MZ (carrier) cohort.

**Methods:**

Individuals with prior MZ results and AAT serum level <16uM were recruited from the Alpha-1 Coded Testing study and Alpha-1 Foundation Research Registry. Participants completed smoking history, demographics, and COPD Severity Score (Range 0-33) using REDCap data capture. At-home finger-stick tests were performed for next generation sequencing (NGS) at the Biocerna LLC laboratory. A genetic counselor reviewed records and interviewed participants with additional variants by NGS. A Wilcoxon Rank Sum test was used to assess correlation between variants and the COPD severity score.

**Results:**

A second* SERPINA1* variant of known or possible significance was identified in 6 (5.8%) participants. One each of ZZ, SZ, FZ, ZSmunich, ZM2obernburg, and Z/c.922G>T genotypes were identified. ZZ, SZ, and FZ are known pathogenic genotypes. Smunich is a likely pathogenic variant. M2obernburg and c.922G>T are variants of uncertain significance. The ZZ individual was on augmentation therapy when determined MZ by protease inhibitor (Pi) phenotyping; the others had limited targeted genotyping with MZ results. These six participants with biallelic variants had positive COPD severity scores >1. Presence of additional variants was not significantly associated with COPD symptoms in this small sample size.

**Conclusions:**

Some diagnosed MZ individuals instead have biallelic variants. Larger studies are needed to determine COPD-risk liability of variants. Accurate diagnosis impacts medical management and familial risk assessment. Pi phenotyping can be confounded by augmentation therapy and liver transplantation. Because a normal M allele may be reported in the absence of tested mutation(s) in AATD genotyping, clinicians should consider clinical circumstances and laboratory methods when selecting and interpreting AATD tests. Advanced testing, including NGS, may be beneficial for select individuals with prior MZ results.

**Clinical Trial Registration:**

This study was registered with clinicaltrials.gov (NCT NCT02810327).

## 1. Introduction

An increased risk for chronic obstructive pulmonary disease (COPD) exists for alpha-1 antitrypsin deficiency (AATD) MZ heterozygotes who have ever smoked [1]. Although excess risk is not established for MZ never-smokers, group mean lung function differences can hide the clinical observation that some MZ individuals present at a young age with panlobular emphysema similar to ZZ (classic, severe) AATD [2]. The genetic and/or environmental contributions to this advanced phenotype are incompletely understood.

Mechanisms to detect genetic signatures that predict clinical phenotypes would allow improved AATD diagnostics and therapeutics. Gene sequencing has not been routinely applied in AATD diagnostics. AATD testing methods commonly include one or more of AAT immunoassay (level), targeted genotyping, and proteinase inhibitor (Pi) phenotyping. The type of AAT testing should reflect the clinical circumstance (targeted detection versus screening) [3]. Conventionally, gene sequencing has been employed only when a rare or null allele is suspected after first tier tests [4].

Over 120 alleles of the AAT-encoding* SERPINA1 *gene have been reported in the literature, and additional unpublished variants have been identified in proprietary laboratories [4, 5]. The Z mutation (c.1096G>A, p.Glu366Lys) is the most common clinically relevant allele, followed by the S mutation (c.863A>T, p.Glu288Val). Together, the S and Z alleles account for >95% of known cases of AATD. Most other alleles are rarely detected and their functional and clinical effects poorly understood. We were interested in using* SERPINA1* next generation sequencing (NGS) to assess whether additional sequence variants impact COPD presence and severity in a previously identified MZ cohort for AATD.

## 2. Methods

**Figure 1:**
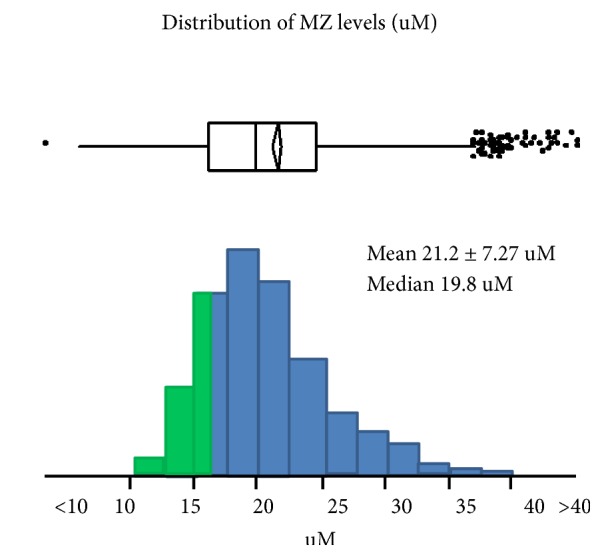
Distribution of AAT levels (uM) among MZ AATD individuals. Those in the lower quartile (less than or equal to 16uM) were included in this study.

Inclusion criteria included age 18 and up and a prior MZ result for AATD with AAT level in the lower quartile for the MZ state (<16uM, 83mg/dL) because additional sequence variants may underlie these lower levels (Figure 1).

Three hundred and seventy eligible individuals from the Alpha-1 Coded Testing (ACT) study and the Alpha-1 Foundation Research Registry were invited to participate by phone and email. The Alpha-1 Coded Testing (ACT) Study is a research study that has tested over 20,000 individuals in the United States and Canada for AATD since 2001 to determine barriers to genetic testing. People with abnormal Alpha-1 genotypes are invited to join the Alpha-1 Foundation Research Registry. Participants in both programs have given permission for recontact for research purposes. This study was listed on clinicaltrials.gov (Registration number: NCT02810327). This research was approved by the Medical University of South Carolina (MUSC) Institutional Review Board.

All participants completed informed consent between June 2014 and June 2015. Following informed consent, participants completed survey questionnaires and reported demographic information, smoking history, and survey-based COPD severity score using a validated integrated measure of disease severity based on symptoms, treatments, and hospitalizations for COPD (range 0-33) [6]. A higher COPD severity score correlates with poorer FEV1 percentage predicted and health related quality of life. The surveys were constructed in REDCap allowing for web-based entry of data [7]. Participants were mailed a home testing kit to perform a finger-stick and submit blood spots for testing under a unique research code. Participants mailed their samples to the Biocerna LLC lab. NGS was performed blinded to the identity and symptomology of the participants with massively parallel high-throughput* SERPINA1* sequencing of coding and promoter regions by the Ion Torrent platform. These bioinformatics suites were used for sequencing and variant interpretation [8].

The genomic interpretations were matched to participant identity by researchers at MUSC and results were mailed to participants. A certified genetic counselor was available for questions. The genetic counselor contacted each participant who had additional findings by NGS. Clinical, laboratory, and patient-reported histories were reviewed to reconcile initial MZ designations with additional NGS findings for these participants.

The MUSC Bioinformatics Core used descriptive statistical analysis to perform means, standard deviations, medians, and percentages and analyzed group genomic data for variant association with COPD severity score using correlation analysis tools focused on keeping a false discovery rate <5%. These analyses included single variant associations and type of variant (e.g., promoter versus coding region) associations with symptoms. Variants found were correlated with symptoms in a Wilcoxon rank sum test using statistical package SAS 9.4 for Windows (SAS Institute Inc., Cary, NC, USA).

## 3. Results

One hundred and seventeen participants were consented (31.6%) and 103 (88.0%) completed the study. Fourteen (12.0%) consented participants did not return their study materials and were excluded from the analyses. Participants were 75.7% female, age range 18-73, and 39.8% ever-smokers. Participants had prior testing through the ACT study (57.3%) and other testing including clinical AATD testing by a clinician. Participant characteristics are shown in Table 1. Six participants (5.8%) had an additional* SERPINA1* sequence variant of known or possible significance. The remaining 94.2% had their MZ genotype confirmed by NGS. Frequency of additional variants did not vary by participant age, gender or smoking history. Each newly identified variant occurred in only one participant and no single variants correlated with COPD symptoms in this small sample size.

One each of ZZ, SZ, FZ, ZSmunich, ZM2obernburg, and Z/c.922G>T (p.Ala308Ser) genotypes were identified. ZZ, SZ, and FZ genotypes are of known clinical significance. Smunich is a likely pathogenic variant. M2obernburg and c.922G>T (p.Ala308Ser) are variants of uncertain significance (VUS) (Table 2).

**Figure 2:**
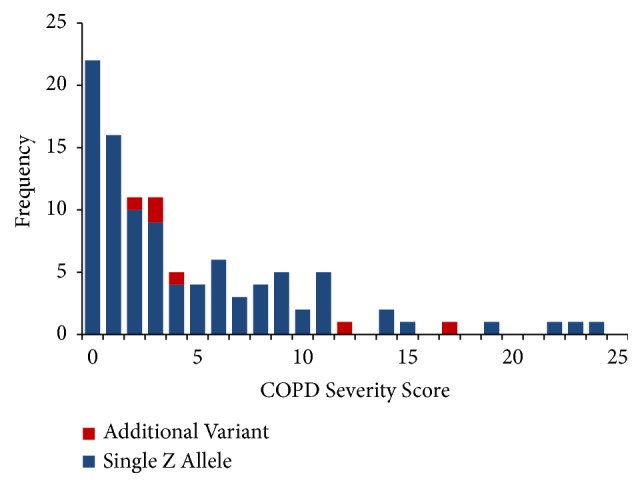
Biallelic variants all had COPD severity scores >1. The distribution of COPD severity scores for all study participants is shown.

All 6 participants with biallelic variants had positive COPD severity scores (>1). The distribution of COPD severity scores for all study participants is shown in Figure 2. The median COPD severity score was 3.0 for confirmed MZs (range = 0-24) in this sample and 3.5 for those with biallelic variants (range = 2-17). These were not statistically significant.

The initial mode of MZ assignment and relevance of new NGS findings are discussed by case below:

### 3.1. Case 1, ZZ

A 59-year-old male was diagnosed with AATD in 1997 by AAT immunoassay (level) and began augmentation therapy in 2014. In 2015, Pi phenotyping yielded Pi MZ results and AAT level of 72mg/dL (13.8 uM). He was identified as a MZ heterozygote and informed his family members of MZ-associated familial risk. Two siblings had genotyping (MM, MZ) and two did not test. The participant received a double lung transplant during the study. The ZZ diagnosis explained his severe, progressive lung disease disproportionate to the MZ genotype. Augmentation therapy was appropriate for severely low pretreatment AAT level and clinical emphysema. The ZZ diagnosis is risk-raising for liver disease where evaluation and monitoring are recommended. The untested siblings have a risk for ZZ-AATD and should be tested.

### 3.2. Case 2, SZ

An 18-year-old male was referred to pulmonology with documented MZ status and AAT level of 64 mg/dL (12.3 uM). The participant's father died of ZZ-AATD disease. He had targeted familial genotyping, which identified a single Z allele as expected, and received consultation about MZ health and reproductive risks. The SZ result by NGS conferred higher health and reproductive risks that require follow-up. Should symptoms worsen in the future, augmentation therapy may be considered (whereas is not recommended for MZ heterozygotes). His results revealed that his mother carries an S allele, and the maternal half-siblings are at previously unknown increased risk.

### 3.3. Case 3, FZ

A 58-year-old female tested through the ACT study in 2014 due to pulmonary symptoms. Prior to 2015 the ACT study performed targeted genotyping for the S and Z mutations only and estimated AAT level. She received a MZ result with AAT level 78 mg/dL (15 uM). The F allele is a dysfunctional allele where the functional capacity to inhibit neutrophil elastase, rather than the quantity of AAT, is altered [9]. The FZ result by NGS diagnosed a rare form of AATD. In the presence of emphysema and fixed obstruction on spirometry, augmentation therapy may be indicated (whereas is not indicated for MZ heterozygotes with the same symptomology). Full siblings have a risk for FZ-AATD and should be tested. Familial testing should cover the F allele to ensure accurate results. Retesting may be recommended for relatives who tested before the F allele was commonly detected. Since 2015, the ACT study genotypes for the Z, S, F, and I mutations.

### 3.4. Case 4, ZSmunich

A 57-year-old male tested through the ACT study in 2016 after his brother was diagnosed with AATD-emphysema. He did not know his brother's genotype. ACT results reported the MZ genotype and level of 70.1 mg/dL (13.6 uM). NGS identified the Smunich variant (c.1061C>T, p.Ser330Phe) which was classified by Biocerna LLC as likely pathogenic [10, 11]. The ZSmunich genotype should be interpreted in the context of AAT level and clinical presentation. This result may alter surveillance and treatment for AATD disease. Genetic risks to relatives are altered by this result although the clinical implications are not well known.

### 3.5. Case 5, ZM2obernburg

A 34-year-old male tested through the ACT study in 2011 and received results with the MZ genotype and level of 80.6 mg/dL (15.5 uM). NGS identified the M2obernburg variant (c.710T>C, p.Gly148Trp) which was classified by Biocerna LLC as a variant of uncertain pathogenicity and uncertain clinical significance [11, 12]. Interpretation of this result in the context of AAT level and clinical symptoms is recommended. Relatives have an increased risk for this variant, although this variant's contribution to AATD risk is unknown.

### 3.6. Case 6, Z/c.922G>T (p.Ala308Ser)

A 65-year-old female tested through the ACT study in 2013 and received results with the MZ genotype and level of 77.0 mg/dL (14.8 uM). NGS identified the c.922G>T (p.Ala308Ser) which was classified by Biocerna LLC as a variant of unknown clinical significance [11]. Interpretation of this result in the context of AAT level and clinical symptoms is recommended. Relatives have an increased risk for this variant, although this variant's contribution to AATD risk is unknown.

## 4. Discussion

Because AATD is a relatively rare condition in individuals who require testing because of COPD or liver disease, testing platforms have been constructed to be most cost effective. In this context, MZ AATD results did not reflect the true genotype in some individuals. Over 5% of previously assessed MZ individuals with AAT level in the lower quartile for the genotype had additional variants that were missed by previous testing. Accurate genotypic diagnosis guides AATD management and genetic risk assessment for family members. Testing limitations, clinical circumstances, and rare variants contribute to inaccurate MZ results. Advanced testing, including NGS, may be beneficial for select individuals with prior MZ results.

AATD testing algorithms and detection rates vary and best testing practice has changed with time [4]. Commercial and institutional labs employ a variety of algorithms, including one or more laboratory methodologies. Guidelines for the diagnosis of AATD (2016) recommend genotyping for at least the S and Z alleles for diagnostic testing of symptomatic individuals [3]. Advanced or confirmatory testing should include Pi-typing, AAT level testing, and/or expanded genotyping. AAT level alone should not be used for the primary diagnosis of AATD. The reasons that people undergo AATD testing are varied and include personal symptoms, family history and general screening. The most limited targeted genotyping detects only the most common clinically relevant Z mutation. Examples include select genetic carrier screens and targeted familial mutation testing. Expanded genotyping detects additional mutations, ranging from S and Z, to 4 or more alleles. The S, Z, F, and I alleles are thought to comprise >99% of AATD mutations, although the frequency and clinical relevance of many less common AATD alleles remains unknown.

AATD allele nomenclature was established based on electrophoretic migration of the alpha-1 proteinase inhibitor (AAT) before the encoding* SERPINA1* gene was identified. This lends to unconventional genomic nomenclature that persists due to wide use and acceptance in the literature. The genotypic designation “M” serves as an alias for the wild-type allele. AATD genotyping tests often report a normal M allele in the assessed genotype, even though genotyping detects specific mutations only and does not confirm a normal allele. One or more M alleles reported by genotyping (e.g., MZ, MM) should more precisely be interpreted as absence of mutation(s) tested. A small but real residual risk exists for patients who have fewer than two mutations identified by genotyping.

Pi phenotyping discriminates serum AAT variants by isoelectric focusing. A normal M protein (including subvariants of M1A, M1V, M2, and M3) and many abnormal AAT variants may be visualized. Pi-type and allelic genotype are the same in most cases and direct inferences can often be made between the two. However, Pi testing for patients on augmentation therapy results in detection of normal M-AAT in serum due to treatment, rather than biologic production.

Augmentation therapy is an FDA approved intravenous AAT replacement therapy indicated for select patients with AATD-emphysema. Normal M-AAT pooled from healthy donor plasma is infused to raise serum AAT levels above the conventionally accepted protective threshold of 11uM. Augmentation slows progression of emphysema in patients with severe deficiency (<11uM) and FEV1 35-70% predicted [13] and has not been extensively studied in other serum level and lung function cohorts. Infused wild-type AAT does not impact DNA level testing for the patient (genotyping or sequencing) but has profound implications for interpretation of Pi phenotyping after therapy initiation, as the normal M-AAT will be detected after therapy as a direct result of the treatment. Serum AAT level will also be elevated as the therapeutic goal. AATD patients who have had a liver transplant have normal M-AAT and AAT level in serum, produced by the transplanted organ. Pi results for patients on augmentation therapy and postliver transplant do not determine the patient's germline genotype. Patient history may not be provided to a testing lab and not all clinicians are aware of these clinical interferences when interpreting results.

Although a majority of individuals with AATD will be ascertained by guideline-based testing, this study shows that some cases are missed. Improved diagnostics and interpretations could reduce error in MZ assignment and improve diagnostic yield. The utility of NGS in AATD diagnostics has not been studied in large series. NGS should be explored in other AATD cohorts and larger studies. NGS permits greater coverage of the* SERPINA1* gene for detection of both common and rare* SERPINA1* variants.

There are some limitations to this study. The individuals in this study were assessed MZ by mixed methodologies (genotyping and Pi-typing) at various points in the past. Therefore, the diagnostic error of any given test is not assessed by this study. Proper application and interpretation of current clinically available methods may reduce the error rate. This study enrolled only individuals with levels in the lower MZ quartile. The diagnostic error rates in MZ individuals with higher levels and individuals tested without a level may be different. The significance of individual variants was not determined by this study. Each additional variant in this study was found in only one participant. Identification of a VUS poses challenges to clinicians and patients/families alike. Variant interpretation is an evolving science. In some cases, laboratories do not agree on assignment of pathogenicity for a single variant (e.g., a VUS at one lab may be considered likely benign at another). Despite these limitations, recognition of sequence variants serves a step toward understanding their prevalence and effect on clinical risk through further research and data aggregation.

In this study NGS identified biallelic variants in over 5% of individuals previously assessed as MZ carriers. This impacts medical management and family risk assessment. These results remind clinicians to consider the clinical circumstances and laboratory methods when selecting and interpreting AATD tests and invite further evaluation of NGS in AATD diagnostics. Advanced or repeat testing, including NGS, may be beneficial for fully defining AATD in select cases.

## Figures and Tables

**Table 1:** Participant characteristics, N= 103.

**Gender**	N (%)	Average Age (range)

Female	78 (75.7)	53.9 ± 12.2 (25-73)

Male	25 (24.3)	46.4 ± 15.9 (18-73)

**Smoking History**		

Ever-smoker	41 (39.8)	

Female	31	53 ± 12.0 (25-71)

Male	10	56 ± 10.7 (34-73)

Never-smoker	62 (60.2)	

Female	47	54 ± 12.1 (27-73)

Male	15	40 ± 14.9 (18-69)

**Recruited From**		

Alpha-1 Coded Testing Study	59 (57.3)	

Alpha-1 Foundation Research Registry or Clinicaltrials.gov	44 (42.7)	

**Table 2:** 

NGS* SERPINA1* Genotype	Variant Significance	Participant Age	Ever- Smoker	COPD Severity Score	AAT level (uM at enrollment)	RepeatAAT level (uM)
ZZ	Pathogenic	59	Yes	17	13.8*∗*	31.1*∗*

SZ	Pathogenic	18	No	2	12.3	9.3

FZ	Pathogenic	58	No	12	15	13

ZSmunich	Likely Pathogenic	67	Yes	4	13.6	11.3

ZM2Obernburg	Uncertain	34	Yes	3	15.5	14

Z/c.922G>T (p.Ala308Ser)	Uncertain	65	No	3	14.8	13

*∗*On augmentation therapy at the time of testing.

## Data Availability

The datasets generated during and/or analyzed during the current study are available from the corresponding author on reasonable request.

## Abbreviations

COPD: Chronic obstructive pulmonary disease
AAT: Alpha-1 antitrypsin
AATD: Alpha-1 antitrypsin deficiency
ACT study: Alpha-1 coded testing study
NGS: Next generation sequencing.
